# Longer lifespan in the Rpd3 and Loco signaling results from the reduced catabolism in young age with noncoding RNA

**DOI:** 10.18632/aging.101744

**Published:** 2019-01-08

**Authors:** Zachary Kopp, Yongkyu Park

**Affiliations:** 1 Department of Cell Biology and Molecular Medicine, Rutgers-New Jersey Medical School, Newark, NJ 07103, USA

**Keywords:** Rpd3 and Loco signaling, reduced catabolism, noncoding RNA, stress resistance, lifespan

## Abstract

Downregulation of Rpd3 (histone deacetylase) or Loco (regulator of G-protein signaling protein) extends *Drosophila* lifespan with higher stress resistance. We found *rpd3*-downregulated long-lived flies genetically interact with *loco*-upregulated short-lived flies in stress resistance and lifespan. Gene expression profiles between those flies revealed that they regulate common target genes in metabolic enzymes and signaling pathways, showing an opposite expression pattern in their contrasting lifespans. Functional analyses of more significantly changed genes indicated that the activities of catabolic enzymes and uptake/storage proteins are reduced in long-lived flies with Rpd3 downregulation. This reduced catabolism exhibited from a young age is considered to be necessary for the resultant longer lifespan of the Rpd3- and Loco-downregulated old flies, which mimics the dietary restriction (DR) effect that extends lifespan in the several species. Inversely, those catabolic activities that break down carbohydrates, lipids, and peptides were high in the short lifespan of Loco-upregulated flies. Long noncoding gene, *dntRL* (CR45923), was also found as a putative target modulated by Rpd3 and Loco for the longevity. Interestingly, this *dntRL* could affect stress resistance and lifespan, suggesting that the *dntRL* lncRNA may be involved in the metabolic mechanism of Rpd3 and Loco signaling.

## Introduction

Rpd3, a *Drosophila* histone deacetylase (HDAC), modulates chromatin structures and affects signaling pathways by interacting with several chromatin remodeling complexes [1-3]. The Rpd3 protein is known to mediate epigenetic effects like long-term memory and lifespan. The adult brain-specific changes of Rpd3 expression results in impaired long-term courtship memory [4]. However, systemic downregulation of Rpd3 extends lifespan [5,6], which is also observed in yeast with reduced Rpd3 expression [7]. Recently, it has been reported that Rpd3 interacts partially with the insulin signaling longevity pathway [8]. As several long-lived mutant flies display increased resistance to numerous stressors including oxidation, starvation, and heat compared to wild-type flies [9-12], Rpd3 downregulation also enhances stress resistance with extended lifespan [6]. Inhibition of mammalian HDACs is shown to be a proven anticancer therapeutic and potential treatment of many other diseases including HIV infection, Alzheimer's disease, and cardiac remodeling [13,14]. Consistently, it was recently reported that decreased Rpd3 expression in *Drosophila* heart tissue enhances cardiac function (decreased heart failure and accelerated heart recovery) and resistance against stressors [6].

Loco, a fly regulator of G-protein signaling (RGS) protein, has been identified as an activator or repressor with GTPase-activating protein (GAP) activity in the G-protein signaling pathway [15,16]. The Loco protein functions in neuroblast (NB) asymmetric division [17] and blood-brain barrier formation [18], which are mediated by interactions with several GPCRs and the inhibitory G proteins (Gαi and Gαo). Beyond these specific cellular functions, it is reported that Loco globally regulates stress resistance and lifespan [19]. Reduced Loco expression results in longer lifespan for flies with stronger resistance to the stressors, higher manganese-containing superoxide dismutase (MnSOD) activity, and higher fat content [19]. In contrast, upregulation of the *loco* gene shortens lifespan significantly with lower stress resistance and reduced fat content [19], also showing that its RGS domain containing GAP activity is related to the regulation of longevity [19]. Notably, the expressional changes of yeast RGS2 and rat RGS14, homologues of the fly Loco, also affect oxidative stress resistance and lifespan in the respective species [19], indicating that the Loco/RGS14 signaling pathway is evolutionarily conserved in various organisms for the regulation of longevity.

To investigate whether the nuclear protein Rpd3 interacts with the cytoplasmic membrane-associated protein Loco for the longevity regulation, here, we measured stress resistance and lifespan with a combination of the *rpd3* downregulation to extend lifespan [5,6] and the *loco* upregulation to shorten lifespan [19]. Then, we examined gene expression profiles between those flies in order to find the common target genes modulated by Rpd3 and Loco for the longevity.

## RESULTS

### Rpd3 and Loco are related to regulate stress resistance and lifespan

**Figure 1:**
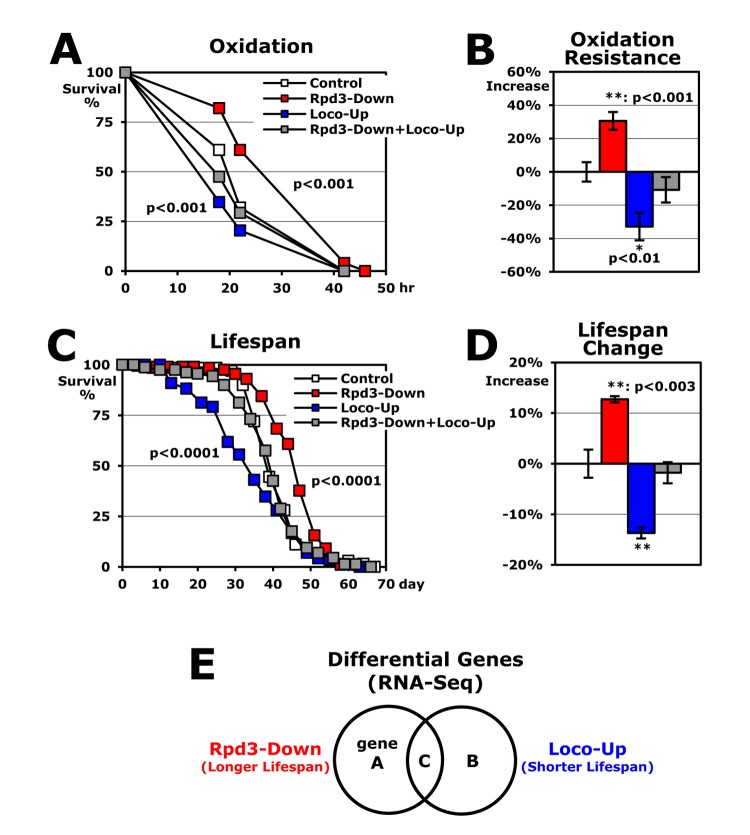
**Rpd3 and Loco regulate stress resistance and lifespan overlappingly.** (**A**) The survival curve for oxidative stress using 2-day-old male flies. Control: UAS-*loco*/+; Rpd3-Down: *rpd3*^-^ UAS-*loco*/+; Loco-Up: UAS-*loco*/actG4; Rpd3-Down+Loco-Up: *rpd3*^-^ UAS-*loco*/actG4; p-value: log-rank test between the control and Rpd3-Down or Loco-Up. (**B**) Changes of oxidative stress resistance. The median survival times of flies under oxidative stress were calculated from several survival curves (**A**) and then the percentage change from control flies (0%) are represented as average ± standard error of mean (SEM) following normalization with the median of control flies (21.4 hours). P-value (*): Student’s t-test. (**C)** The lifespan of adult male flies. (**D**) Percent changes of mean lifespan are indicated as average ± SEM normalized by the control’s mean lifespan (UAS-*loco*/+: 39.4 days), which were calculated from several lifespan curves (**C**) of 2 ~ 4 independent experiments. The mean lifespan of another control +/actG4 flies was 40.1 days as single transgenic. (**E**) Venn diagram of differential genes with RNA-seq analyses between the control and Rpd3-Down or Loco-Up. Gene A and B indicate the groups changed with Rpd3-Down and Loco-Up, respectively. The C represents commonly changed genes in both Rpd3-Down and Loco-Up.

When the *rpd3* heterozygous (P{PZ}HDAC1^04556^/+) mutant flies [5] were incubated with paraquat-induced oxidative stress, this *rpd3* downregulation (Rpd3-Down: *rpd3*^-^ UAS-*loco*/+) enhanced stress resistance up to 30% from the control (UAS-*loco*/+) flies (Figure 1A-B) [6]. Using an UAS/Gal4 system [20], next we upregulated the genomic *loco* gene containing an UAS region in the promoter (P{XP}loco^d06164^) with an *act*-Gal4 driver that expresses a target UAS-gene in the whole body. In contrast to the higher stress resistance shown with the *rpd3* downregulation (Figure 1A-B), the *loco* upregulation (Loco-Up: UAS-*loco*/actG4) reduced survivorship by 33% under oxidative stress compared to the common control (UAS-*loco*/+ in Figure 1A-B) [19]. When *rpd3* downregulation and *loco* upregulation were combined (Rpd3-Down+Loco-Up: *rpd3*^-^ UAS-*loco*/actG4), interestingly, the oxidative stress resistance showed an intermediate level (-11% in Figure 1A-B), which suggests that there is an interaction between the Rpd3 and Loco phenotypes in the stress resistance. The lifespans of Rpd3-Down, Loco-Up, and Rpd3-Down+Loco-Up flies (Figure 1C-D) also exhibited a similar pattern as shown in assessing stress resistance (Figure 1A-B), implying that the Rpd3 and Loco probably regulate longevity with common downstream genes or pathways.

To find the target genes that both Loco and Rpd3 modulate for stress resistance and lifespan, we examined gene expression profiles of the *rpd3* downregulation (Rpd3-Down: *rpd3*^-^UAS-*loco*/+) or the *loco* upregulation (Loco-Up: UAS-*loco*/actG4) versus the common control (UAS-*loco*/+) flies, independently (Figure 1E). We focused on the "C" group (Figure 1E) showing an opposite pattern in the expression level between the Rpd3-Down (longer lifespan) and Loco-Up (shorter lifespan) groups because the "C" genes would be changed in two different longevity phenotypes (Figure 1A-D).

### Longer lifespan enhanced by changes in Rpd3 and Loco exhibits the reduced catabolism

**Figure 2:**
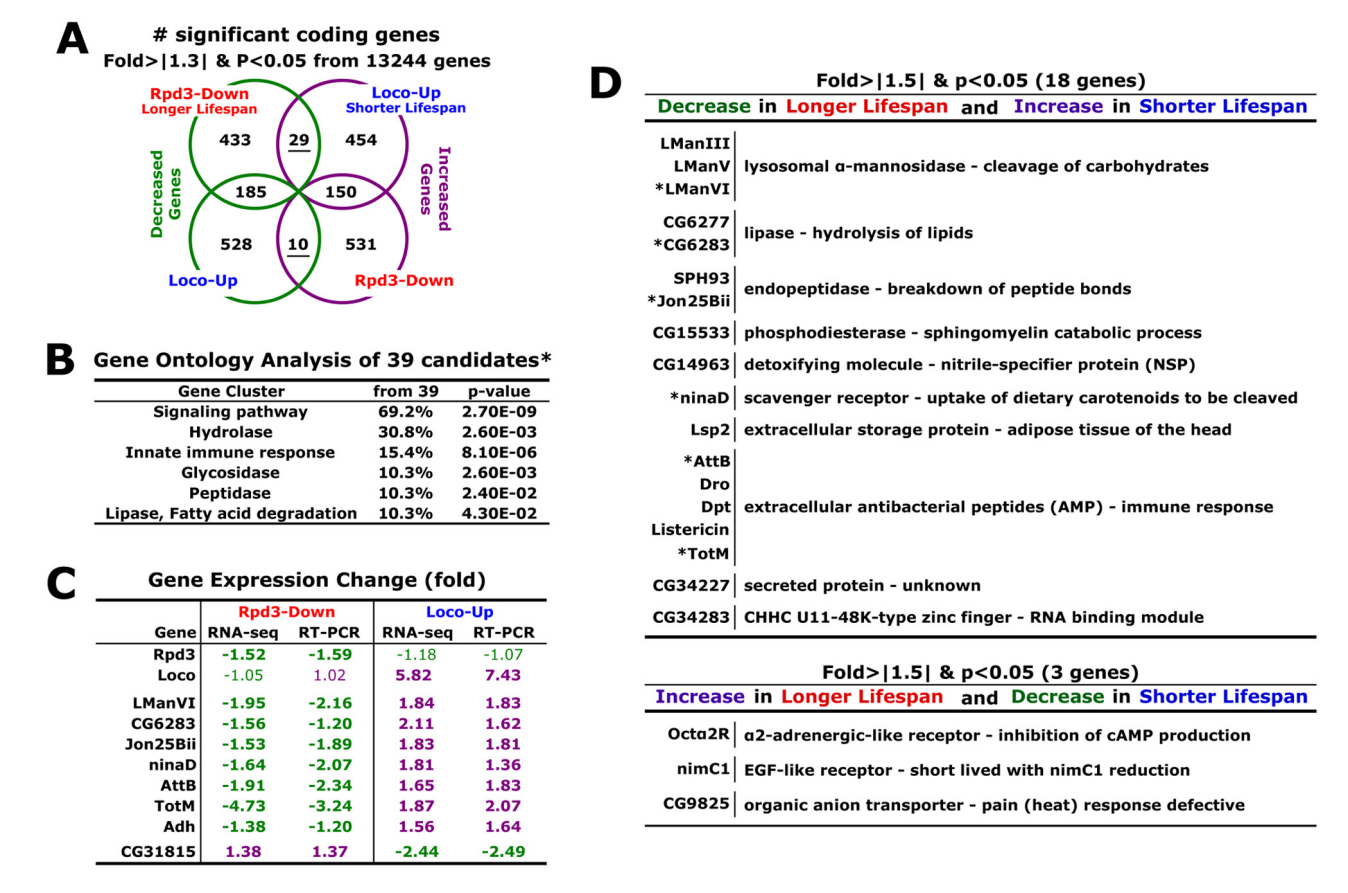
**Longer lifespan induced by the change of Rpd3 and Loco results from the reduced catabolism.** (**A**) The gene expression analyses (Rpd3-Down or Loco-Up/control). From RNA-seq experiments using 2-day-old male flies, the genes changed more than 1.3-fold were selected with p-value <0.05 (Fisher’s exact test). The underlined genes represent that their expressions decrease (green) and increase (purple) in a different group, respectively. (**B**) With the genes oppositely changed in the Rpd3-Down and Loco-Up (*: total 39 in **A**), the gene ontology was analyzed using a DAVID web tool (http://david.abcc.ncifcrf.gov/home.jsp). %: involved genes/total 39 genes; p-value: a modified Fisher Exact. (**C**) Expressional changes of selected genes in RNA-seq and real-time PCR analyses. The fold changes were averaged from 4 ~ 13 independent experiments using four different RNA batches. Bold change: p-value < 0.05 ~ 0.0001. (**D**) Functional analyses (http://flybase.org) of genes which were selected with more than 1.5-fold changes and p-value <0.05 in both Rpd3-Down (longer lifespan) and Loco-Up (shorter lifespan) groups. *: genes tested as a representative of each functional subgroup with RT-PCR analysis in (**C**).

With a total of 49,162,435 reads of RNA-seq experiments, RNA expression levels of the 13,244 genes were compared between 2-day-old male flies of experimental and control groups (Rpd3-Down/ or Loco-Up/common control). First, we statistically filtered out the genes that are non-specifically changed in the expression with a p-value < 0.05 (Fisher’s exact test). Then, with a cut-off value of ± 1.3-fold changes, we selected the candidates as significantly changed genes (Figure 2A). In the *rpd3*-downregulated flies (Rpd3-Down), 647 genes showed decreased expressions and 633 gene expressions were increased with the *loco* upregulation (Loco-Up) (Figure 2A). From these two gene groups, only 29 genes were overlapped reflecting the opposite expression between the longer lifespan (Rpd3-Down) and shorter lifespan (Loco-Up) (Figure 2A). This indicates that the 29 expressions decrease in the long-lived flies with Rpd3 downregulation and conversely increase in the short lifespan of Loco-upregulated flies. Reverse analysis (Figure 2A) revealed that 10 genes show an expression pattern of an increase in the longer lifespan and a decrease in the shorter lifespan. Collectively, these 39 total genes (~ 0.3% from 13,244 genes initially screened) were considered as candidate target genes (Figure 2A) which both Loco and Rpd3 modulate for stress resistance and lifespan (Figure 1).

When the gene ontology of these 39 genes was analyzed with a DAVID web tool (Figure 2B), 69% of them were shown to be related to the signaling pathways. Interestingly, many of them are metabolic enzymes, suggesting that Loco and Rpd3 change the metabolism for the longevity. Through RT-PCR analysis (Figure 2C), we confirmed expression changes of candidate genes obtained from the RNA-seq experiments (Figure 2A). As representatives of each functional subgroup in Figure 2D, they showed opposite expressions in the longer lifespan (Rpd3-Down) and shorter lifespan (Loco-Up), respectively (Figure 2C). However, the Rpd3 and Loco did not affect expressions of each other. Among the 39 candidate genes (Figure 2A), we next examined more significantly changed genes with more than 1.5-fold changes [21-23] in both Rpd3-Down and Loco-Up groups (Figure 2D). Interestingly, the activities of catabolic enzymes such as mannosidase, lipase, endopeptidase, and phosphodiesterase [24] were reduced in the long-lived flies with Rpd3 downregulation (Figure 2D). Uptake and storage proteins (ninaD and Lsp2) of diets to be catabolized [25,26] were also decreased in the flies with longer lifespan (Figure 2D). Inversely, those catabolic activities that break down carbohydrates, lipids, and peptides were high in the short lifespan of Loco-upregulated flies (Figure 2D). Other notable changes were an apparent decrease in the detoxifying molecule and antibacterial peptides (AMP) in the longer lifespan of Rpd3 downregulation (Figure 2D). As defending mechanisms, these proteins are essential to protect flies against toxins and bacterial infections [27-29], and the AMP expressions increase during the aging process [22], which may prepare pathogen defenses promptly in the old-aged flies that have weak immune responses. However, it has been reported that lesser expressions of AMP genes are found in longer-lived flies [22] and the AMP downregulation enhances stress resistance, lifespan, and fat content in adult flies [30]. Lifespan extension through hormesis (the beneficial effects of low-level toxins and stressors) also occurs with reduced immune function [31]. These data imply that less activation of AMP synthesis has a beneficial effect for the longevity process under the normal condition without bacterial infection. Consistent with this hypothesis, higher expressions of AMP genes were detected in the short lifespan of Loco-upregulated flies (Figure 2D).

On the contrary, expressions of Octα2R, nimC1, and CG9825 genes were increased in the long-lived flies under Rpd3 downregulation (Figure 2D). Octα2R (α2-adrenergic-like octopamine receptor) is activated by octopamine and epinephrine, resulting in the inhibition of cAMP production in a dose-dependent manner [32]. The cAMP reduction has been observed in several long-lived animals such as AC5 (type 5 adenylyl cyclase) KO mice and Loco (Regulator of G-protein signaling protein) mutant flies [19,33]. It was reported that reduction of nimC1 (EGF-like receptor) makes flies live for a shorter time [34] and the downregulation of the CG9825 gene (organic anion transporter) is defective in the pain (heat) response [35]. Consistently, the decrease of nimC1 and CG9825 expressions were shown in the short-lived flies with Loco upregulation (Figure 2D).

### Noncoding RNAs are related to the longevity of Rpd3 and Loco

**Figure 3:**
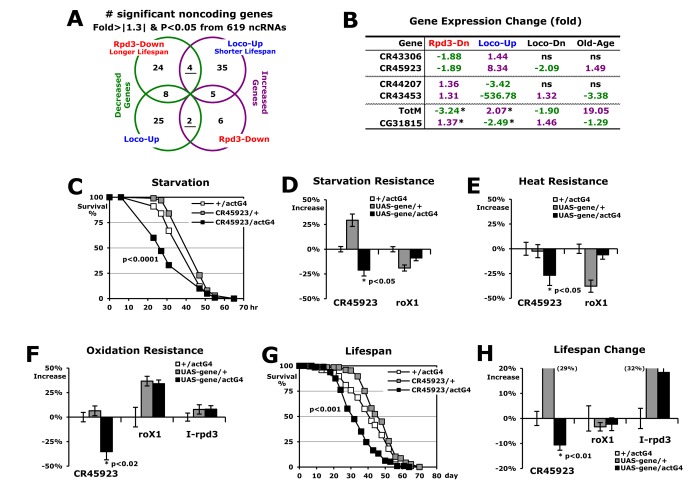
**Noncoding RNAs related to the longevity of Rpd3 and Loco.** (**A**) Expression analyses of the noncoding genes from RNA-seq experiments of Rpd3-Down or Loco-Up over the control. Among the genes with fold change > |1.3| and p-value < 0.05, the underline indicates that the expression decreases (green) and increases (purple) in each different group. (**B**) Expressional fold changes of selected genes with real-time PCR analyses (p-value < 0.02 ~ 0.0001). Loco-Dn: comparison between 2-day-old male flies of *loco*^-^/+ and wild-type (+/+); Old-Age: expression in the old flies (7 weeks) over young (1 week) flies between wild-type males; ns: non-specific change; *: expression changes of coding genes tested in Figure 2D. (**C**) The survival curve for starvation stress using 2-day-old male flies. P-value: log-rank test between the control (+/actG4) and CR45923/actG4 flies. (**D**-**F**) Stress resistance changes against starvation (**D**), heat (**E**), and oxidation (**F**). The percentages of median survival times changed from the +/actG4 flies (0%) are represented as average ± SEM followed by calculation of the median from several stress survival curves. P-value (*): Student’s t-test; I-rpd3: inverted sequence of rpd3. (**G**) The lifespan of adult male CR45923/actG4 flies with the controls (+/actG4 and CR45923/+). (**H**) Percent changes of mean lifespan are indicated as average ± SEM normalized by the +/actG4’s mean lifespan (39.3 ~ 42.8 days), which were calculated from several lifespan curves (**G**) of 3 ~ 5 independent experiments and were also tested with another driver *tublin*-Gal4. Parentheses: changed percentage out of data range in the graph.

Using ribosomal RNA-depleted total RNA in the RNA-seq experiments, we examined whether noncoding RNAs are involved in stress resistance and lifespan regulated by Rpd3 and Loco. With a cut-off value of ± 1.3-fold changes and a p-value < 0.05 (Fisher’s exact test), we found that six total noncoding genes exhibit opposite expressions between the *rpd3*-downregulated long-lived flies and the *loco*-upregulated short-lived flies (four or two genes in Figure 3A). By RT-PCR analysis (Figure 3B), we confirmed expression changes of each of the two candidate genes from the different noncoding groups (Figure 3A). When their expressions were examined in long-lived flies with Loco downregulation [19], especially, the CR45923 and CR43453 genes showed the opposite changes compared to the *loco*-upregulated short-lived flies (Figure 3B), indicating that these two noncoding genes are specifically dependent on the Loco change to regulate longevity. Interestingly, their expressions were changed in old age as shown in the *loco*-upregulated short-lived flies (Figure 3B). Considering that Loco expression increases during the aging process [19], the expressions of CR45923 and CR43453 in old age may be affected by Loco upregulation, which probably contributes to the acceleration of the aging process. As representatives of coding genes in Figure 2C, the TotM and CG31815 genes also exhibited the comparative expression pattern in Loco-Dn and Old-Age from the Loco-Up (Figure 3B).

Next, we investigated if the noncoding CR45923 RNA, which is modulated by Rpd3 and Loco for longevity (Figure 3B), can affect stress resistance and lifespan. Interestingly, the CR45923-upregulated (UAS-*CR45923*/actG4) flies survived significantly less under the starvation stress than did two control (+/actG4 and UAS-*CR45923*/+) flies (Figure 3C), and the median survival time was lower by 21% (Figure 3D). It is consistent with the report that the Loco-upregulation (UAS-*loco*/actG4), which increases the CR45923 expression (Figure 3B), also causes a lower survival rate in response to starvation stress [19]. Similarly, during the other stress (heat and oxidation) tests, the UAS-*CR45923*/actG4 flies exhibited less survivorship than the control flies, showing 24% and 35% decreases under heat and oxidative stress, respectively (Figure 3E-F). To test whether any noncoding RNA overexpression induces a negative effect on the stress resistance, we upregulated the *roX1* noncoding RNA that functions in dosage compensation of the *Drosophila* male X chromosome [36]. However, the roX1-upregulated (UAS-*roX1*/actG4) flies did not show a significant change in survivorship from the three stressors compared to the two control (+/actG4 and UAS-*roX1*/+) flies (Figure 3D-F). Overexpression of another noncoding RNA, I-rpd3, to produce a non-specific RNA of inversed *rpd3* gene, still did not change the stress resistance of flies (Figure 3F). Then, it was shown that the lifespan of CR45923-upregulated flies was significantly shortened by 14% (Figure 3G-H), compared to no effect of roX1 and I-rpd3 upregulation on the lifespan of flies (Figure 3H). These results demonstrate that the noncoding CR45923 RNA is modulated as a downstream noncoding target of Rpd3 and Loco for the longevity regulation (Figure 3B), which is hereby referred to as the ‘dntRL’ gene. Consistently, expression of this *dntRL* gene was not changed in the flies in which lifespan was not affected, such as I-rpd3 or rpd3WT-upregulated flies (data not shown).

## DISCUSSION

Rpd3 or Loco downregulation extends lifespan with higher stress resistance [5,6,19]. Here, we found that the nuclear protein Rpd3 genetically interacts with the cytoplasmic membrane-associated protein Loco in the longevity mechanism (Figure 1A-D). Given that the changes in Rpd3 and Loco did not affect the expression of each other (Figure 2C), they may interact post-translationally through a signaling pathway. As a regulator of G-protein signaling protein, Loco increases adenylate cyclase (AC) activity by inactivating the inhibitory Gαi•GTP protein [17,18,33], and the mammalian homologue, RGS14, interacts with activated H-Ras and Raf-1 kinases, which subsequently inhibit ERK phosphorylation [37-39]. Consistently, we previously showed that downregulation of Loco significantly diminishes cAMP amounts [19] and increases p-ERK levels with higher resistance to the oxidative stress [12]. In mammals, p-ERK is shown to interact with PP1 (protein phosphatase 1) that dephosphorylates p-HDAC1 [40-42]. Considering mammalian HDAC1 phosphorylation promotes complex formation and enhances the enzyme activity of HDAC1 [42-44], one possibility is that Loco downregulation may decrease Rpd3 activity through the reduction of phospho-Rpd3 levels, subsequently resulting in the longer lifespan (Figure 1).

A second possibility of interaction between Rpd3 and Loco is that they regulate the metabolic pathways that converge to impact longevity (Figure 2). When we examined the target genes showing opposite expression between the *rpd3*-downregulated long-lived flies and the *loco*-upregulated short-lived flies (Figure 2A-C), we found that the expressions of catabolism process genes were significantly reduced in the long-lived flies (Figure 2D). Given that these gene expressions were analyzed in young flies (2 days of age in Figure 2A), the reduced catabolism is considered to be necessary from a young age for the longer lifespan of the Rpd3- and Loco-downregulated old flies (Figure 2D). This reduced catabolism is related to the dietary restriction (DR) that extends lifespan in several species [39]. Also, it was recently reported that Rpd3 interacts partially with the insulin signaling pathway of the DR longevity mechanism [8,45].

The noncoding RNA, *dntRL* (CR45923), was found to be a putative target gene that is modulated by Rpd3 and Loco for the longevity (Figure 3B). Surprisingly, this RNA itself could affect stress resistance and lifespan (Figure 3C-H). The fly database (http://flybase.org) indicates that the *dntRL* gene is expressed in fat body tissue, which functions in metabolic homeostasis, stress tolerance, growth, and longevity in *Drosophila* [46,47]. The antimicrobial peptides (AMPs) genes are also dominantly expressed in the fat body [27] as other target genes modulated by Rpd3 and Loco for the longevity (Figure 3B). Together with a report that the *dntRL* gene was screened during a mitochondrial disease-related study [48], it is considered that the *dntRL* gene may be involved in the metabolic mechanism as a long noncoding RNA (1,364 nt).

## MATERIALS AND METHODS

### Fly genotypes and constructs

The *y^1^ w^1^* (lab stock from Bloomington) flies were used as a wild-type background. The *rpd3*^-^ (P{PZ}HDAC1^04556^: Bloomington), UAS-*loco* (P{XP}loco^d06164^: Exelixis at Harvard Medical School), and *act*-Gal4 (Bloomington) flies were obtained from the *Drosophila* stock center. The *loco*^-^ (*loco^P283^*: W. Chia [17],) and UAS-*roX1* (RL. Kelley [49],) were kindly provided. These flies were six times isogenized with *y^1^ w^1^* before the stress resistance and aging tests. Virgin flies were collected from a bottle in which larval density was controlled in a standard cornmeal medium, and were used for all fly experiments including stress response, aging, and gene expression studies [19]. To construct UAS-*CR45923* and UAS-I-*rpd3* transgenes (Figure 3), the *CR45923* (1,364 bp) and *rpd3* (1,563 bp) cDNAs were cloned into the pCRII-TOPO vector (Invitrogen). After the sequences were confirmed, the plasmids were subcloned into an EcoRI/XhoI or XhoI/XbaI digested pUASTattB vector.

### Stress response and aging assays

To measure stress responses, 100 newly enclosed flies (20 flies per vial) were kept in a standard cornmeal medium at 25°C for 2 days [9,19,50]. For the heat test, these 2-day-old adult flies were transferred to new vials containing standard cornmeal medium and maintained at 37°C with 30% humidity. For the starvation test, the 2-day-old adult flies were transferred to new vials (2.5 × 9.3 cm) containing two filter circles (2.4-cm diameter, Fisher Scientific) soaked in 300 μl of distilled water, and were maintained at 25°C under moist conditions with 100 μl of water added every 12 hrs. For the oxidative stress test, the 2-day-old adult flies were starved for 6 hrs at 25°C as described above. Then, the flies were transferred to new vials containing two filter circles wetted with 300 μl of 20 mM methyl viologen hydrate (Paraquat, Fisher Scientific) in a 5% sucrose solution and maintained at 25°C. The median survival times of flies under each stress (heat, starvation, or oxidation) were calculated from the survival curves of 3 ~ 6 independent experiments. For the aging test, 200 virgin flies (20 flies per vial) were counted and transferred to fresh standard cornmeal vials every 3 - 4 days [9]. Mean lifespan was calculated from the lifespan curves and averaged with standard error of mean (SEM) from several repeat experiments.

### RNA-seq and RT-PCR analyses

Total RNAs were extracted with the TRIzol Reagent (Invitrogen) from the whole body of male adult flies and then ribosomal RNAs were depleted from the total RNAs using the RiboMinus Eukaryote Kit (Invitrogen). With these coding and noncoding RNAs, the Illumina compatible strand-specific RNA-Seq protocol was used to make the cDNA library, which was then sequenced by Illumina NextSeq500 sequencer in the Genomics facility (http://research.njms.rutgers.edu/genomics/). The significance of the expression changes was evaluated by the Fisher’s exact test (p-value). For the RT-PCR experiment to check transcriptional expression of the genes, 5 μg of the total RNA purified from adult flies were treated with DNase I (RNase-free, Roche) and used to produce oligo dT-primed cDNAs (SuperScript II RT, Invitrogen). Then, the cDNAs were used as templates for quantitative real-time PCR that was performed with power SYBR green PCR mix (Applied Biosystems) [51]. The *rp49* gene was used as an internal reference for normalizing the quality of total RNA purified from each fly. Expressional fold of the various genes was determined by the comparative C_T_ method (ABI Prism 7700 Sequence Detection System User Bulletin #2, Applied Biosystems).
